# Phase 1 study to evaluate safety, tolerability and pharmacokinetics of a novel intra-tympanic administered thiosulfate to prevent cisplatin-induced hearing loss in cancer patients

**DOI:** 10.1007/s10637-020-00918-1

**Published:** 2020-03-10

**Authors:** Vissia Viglietta, Fuxin Shi, Qi-Ying Hu, Yong Ren, John Keilty, Heather Wolff, Ryan McCarthy, Jason Kropp, Pete Weber, John Soglia

**Affiliations:** Decibel Therapeutics, 1325 Boylston Street, Suite 500, Boston, MA 02215 USA

**Keywords:** Ototoxicity, Hearing loss, Cisplatin, Intratympanic

## Abstract

Cisplatin is a widely used chemotherapy for the treatment of certain solid tumors. Ototoxicity and subsequent permanent hearing loss remain a serious dose-limiting side effect associated with cisplatin treatment. To date, no therapies have been approved to prevent or treat cisplatin-induced hearing loss (CIHL). Sodium thiosulfate effectively inactivates cisplatin through covalent binding and may provide protection against cisplatin-induced ototoxicity. DB-020 is being developed as a novel formulation of sodium thiosulfate pentahydrate in 1% sodium hyaluronate for intratympanic injection (IT), enabling the delivery of high concentrations of thiosulfate into the cochlea prior to cisplatin administration. In the DB-020-002 phase 1a single-ascending dose study, healthy volunteers were enrolled into 5 cohorts to receive different doses of DB-020 via IT injection. Cohorts 1–4 received unilateral injections while Cohort 5 received bilateral injections. Plasma thiosulfate pharmacokinetics was measured, and safety and audiometric data were collected throughout the study. This study has demonstrated that intratympanic administration of DB-020 results in nominal systemic increases in thiosulfate levels, hence it should not compromise cisplatin anti-tumor efficacy. Furthermore, DB-020 was safe and well tolerated with most adverse events reported as transient, of mild-to-moderate severity and related to the IT administration procedure. These results support the design and execution of the ongoing proof-of-concept study, DB-020-002, to assess otoprotection using DB-020 in cancer patients receiving cisplatin without negatively impacting cisplatin anti-tumor efficacy.

## Introduction

Cisplatin is a widely used and effective chemotherapy for the treatment of adult and pediatric solid tumors, including bladder, testicular, head and neck, and lung cancers [1]. Serious side effects of cisplatin treatment include neurotoxicity, nephrotoxicity and ototoxicity often leading to permanent hearing loss. To date, no approved therapy to prevent or treat ototoxicity exists for patients receiving cisplatin treatment, which remains a major dose-limiting side effect of cisplatin administration [2].

In multiple animal models, cisplatin-induced hearing loss has been shown to result from the death of specialized sensory outer hair cells (OHCs) that amplify sound in the cochlea [3, 4]. The incidence of clinically significant hearing loss in patients receiving cisplatin is correlated with cumulative dose [5]. Outer hair cell degeneration induced by cisplatin is more severe at the base of the cochlea (region responding to high frequency sound stimulation) and it progresses to the apex affecting lower frequencies. Cisplatin also damages inner hair cells, spiral ganglion, and stria vascularis [1, 2]. A recent study of temporal bones from patients demonstrated that cisplatin is retained indefinitely in the cochlea [6]. Cisplatin-induced hearing loss generally manifests as irreversible, progressive, bilateral, high frequency sensorineural hearing loss with tinnitus. Tinnitus may occur with or without hearing loss and may be permanent or transient [1].

Thiosulfate is a strong nucleophile binding directly to cisplatin [7, 8]. The rate of reaction between thiosulfate and cisplatin is concentration-dependent and at high molar ratios (thiosulfate to cisplatin), reaction rates are on the order of minutes [9]. The binding of thiosulfate to cisplatin is generally non-reversible and renders cisplatin nontoxic. Therefore, delivering high concentration of thiosulfate to the cochlea at the time of cisplatin administration, will likely result in complete detoxification of cisplatin and consequent protection of hair cells and hearing.

Studies have shown systemic administration of thiosulfate to provide moderate levels of hearing protection [3, 10] but this requires high intravenous thiosulfate dose (20 g/m^2^). This results in high systemic levels of thiosulfate (≈ 3–4 mM), requiring thiosulfate to be delivered a minimum of six hours post-cisplatin administration to alleviate negative impact on cisplatin efficacy. The unintended consequence in this study was a lower event-free survival and lower overall survival for patients treated with systemic thiosulfate compared to the control group (10) which underscores the need for intratympanic administration of thiosulfate to prevent such harmful complications.

Intra-tympanic (IT) administration is a common technique used by otolaryngologists to deliver high concentration of drugs to the auditory and vestibular systems. The use of IT administration to selectively administer thiosulfate to the inner ear provides the opportunity to minimize systemic exposure thus avoiding a negative impact on cisplatin efficacy. IT administration of a thiosulfate containing gel has been evaluated in a Phase 2 clinical study to examine hearing protection in patients receiving cisplatin for head and neck cancers [11]. In the study, IT administered thiosulfate was safe, well tolerated, and showed trends towards clinical efficacy. It is uncertain whether protective levels of thiosulfate were present in the cochlea at the time of cisplatin dosing in the study as thiosulfate gel was administered the day before patients received cisplatin.

**Fig. 1:**
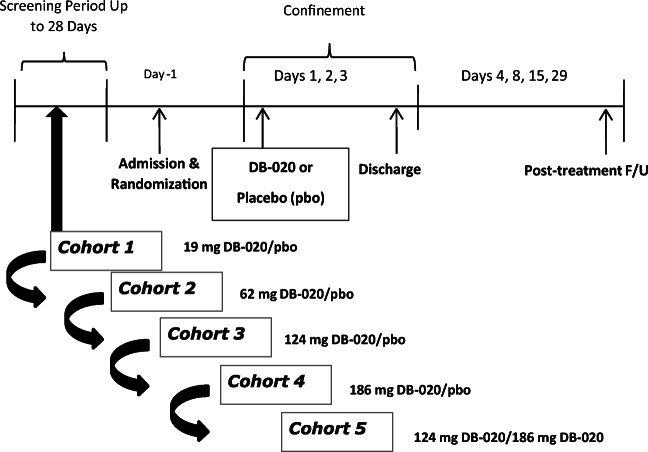
Study Schema. Cohorts 1–4 included 8 subjects randomly assigned (3:1) to receive either DB-020 (6 subjects) or placebo (2 subjects). The ear to be injected was randomly assigned. Subjects in Cohorts 1–4 received a unilateral injection. Subjects in Cohort 5 received bilateral injections (both ears receiving the same study drug as randomized) and 10 subjects were enrolled (1 subject received placebo). The dose in Cohort 5 was selected following review of safety and tolerability observed in the unilateral cohorts

IT administration of thiosulfate has been evaluated in pre-clinical studies to show that complete protection from cisplatin-induced hearing loss is possible when thiosulfate is administered prior to cisplatin treatment [12]. DB-020 is a novel formulation of sodium thiosulfate pentahydrate in sodium hyaluronate. IT studies conducted in guinea pigs have demonstrated cochlear dose levels greater than 0.62 to 2.48 mg/ear (6 to 25% *w*/*v*, 0.25 to 1 M) provide complete protection from cisplatin ototoxicity when administered 3 h prior to cisplatin administration [13]. Preclinical DB-020 data supported the design of a Phase1 clinical study (Fig. 1) aiming to evaluate the safety and tolerability of DB-020 IT administration as well as to collect extensive systemic thiosulfate pharmacokinetics measurements to enable evaluation of potential impact to cisplatin efficacy in humans.

In this article we report safety and pharmacokinetic (PK) results from a Phase1 study in healthy volunteers who received DB-020 via IT administration. Results show only nominal increases in plasma thiosulfate levels that, at Cmax, are far below the levels thought to interfere with cisplatin levels, indicating DB-020 IT administration should not impact cisplatin efficacy. DB-020 was also shown to be safe and well tolerated. The totality of scientific evidence and acceptable safety profile of DB-020 has led to the initiation of a proof of concept study in cancer patients receiving cisplatin.

## Methods

### Study population

This was a Phase1 randomized, double blind, placebo controlled single ascending-dose study to evaluate safety, tolerability and pharmacokinetics of DB-020 conducted in healthy volunteers. This study was performed at a single study center (CMAX Clinical Research, Level 5, 18a North Terrace, Adelaide SA 5000, Australia).

42 healthy men (*n* = 17, 40%) and women (*n* = 25, 60%) aged 19 to 47 years, were enrolled into 5 cohorts. Subjects must not have had any clinically significant active or chronic medical conditions, must have had normal laboratory values (hematology, serum chemistry, urinalysis, and serology) and normal otoscopic findings in both ears and a hearing threshold of 25 dB or better in both ears as assessed with audiometry. Cohorts 1–4 included 8 subjects randomly assigned in a 3:1 ratio to receive either DB-020 (6 subjects) or placebo (2 subjects) to one ear only (unilateral). The ear to receive the IT injection was randomly assigned. Cohort 5 included 10 subjects: 9 randomized to receive DB-020 and 1 randomized to receive placebo to both ears (one DB-020 subject chose to receive only the first injection). Only the site pharmacist was unblinded to study treatment; all other Site staff, sponsor personnel, and the subjects were blinded to the study treatment assigned for Cohorts 1–4 and the sentinel subjects in Cohort 5.

### Intratympanic administration of DB-020

The dose levels per cohort were as follows; Cohort 1: 3.7% *w*/*v* (0.15 M), Cohort 2: 12% w/v (0.5 M), Cohort 3: 25% w/v (1.0 M), Cohort 4: 37% w/v (1.5 M), Cohort 5 bilateral: 37% w/v and 25% w/v (Table 3). Treatment was administered by otologists via IT injection. Lidocaine was injected locally to subjects in Cohorts 1–4 and all but 4 subjects in Cohort 5. These 5 subjects received topical EMLA® cream. Subjects were positioned on their side with the ear to be injected facing up. DB-020 was injected in the central posterior inferior quadrant over the round window. DB-020 coverage of the round window areas was ascertained visually. Subjects remained positioned with injected ear facing up and were instructed to refrain from talking, swallowing, ear popping, yawning, and blowing their nose for 15–30 min. In the bilateral cohort, subjects turned their head after 15–30 min and the procedure was repeated in the contralateral ear.

### Human plasma thiosulfate pharmacokinetic sample collection and processing

Serial blood samples (~2 mL) to measure thiosulfate plasma concentration were collected at the following timepoints: pre-dose (~15 min before dosing) and at 0.25, 0.5, 1, 2, 3, 4, 6, 8, 10, 12, 16, 24, 36, and 48 h after study-drug administration. Additional plasma PK sampling obtained at 72 (±2 h) hours post-dose, on days 8 (168 h), 15 (336 h) and 29 (672 h) post-dose. Blood was collected into K2EDTA collection tubes. Tubes were gently inverted and kept in chilled water bath (4C) until centrifugation at 1500gat, 4C for 10 min. Plasma was transferred into polypropylene tubes and frozen promptly. Samples were stored at -80C until analyzed by liquid chromatography tandem mass spectrometry (LC-MS/MS).

#### Tympanometry

Tympanometry measures the mobility of the middle ear system. Tympanometry was administered by an audiologist using a calibrated Interacoustics Titan, a commercially available diagnostic middle ear analyzer.

#### Pure tone thresholds (audiogram)

Pure tone air and bone conduction audiometry was administered with a calibrated commercially available audiometer, the Interacoustics Affinity 2.0. Pure tone thresholds were measured by an audiologist while the subject was seated in a sound booth with ambient noise levels measured below the maximum permissible ambient noise levels (MPANL) criteria per ANSI S3.1–1999 (R2008) [14, 15]. Pure tone bone conduction audiometry was measured from 250 to 4000 Hz with a B71 bone transducer headset. Pure tone air conduction thresholds were measured with circum-aural headphones and collected at the conventional frequency range (250-8000 Hz) and at the extended high frequencies (9000–16,000 Hz). Measuring the hearing thresholds at the extended high frequency range provides evidence of ototoxicity before any hearing loss is detected by conventional systems [16].

#### Distortion product Otoacoustic emissions

Distortion product otoacoustic emissions (DPOAEs) serve as an objective measure of outer hair cell (OHC) function [17]. DPOAEs were measured by an audiologist using the calibrated Interacoustics Titan from 1000 to 8000 Hz at soft to moderate levels to assess the response of the OHCs at different regions of the inner ear.

#### Tinnitus functional index

The Tinnitus Functional Index (TFI) is a standard self-report tinnitus questionnaire used for evaluating the functional effects of tinnitus at intake assessments, and for measuring intervention-related changes. The TFI consists of a set of 25 questions that lead to an overall score and the possibility to examine 6 subscales which assess different aspects of tinnitus (intrusiveness, sense of control, cognitive, sleep, auditory, relaxation, quality of life, and emotional).

#### Hearing handicap inventory for adults

The Hearing Handicap Inventory for Adults (HHIA) is a standard self-report questionnaire of an individual’s reaction to their hearing loss. The HHIA is a set of 25 questions focusing on the social and emotional impact of hearing loss. The HHIA is a widely used and validated instrument [18].

### Statistics

All data were summarized using descriptive statistics and placebo groups were pooled across cohorts. Continuous data included number of subjects (n), mean, standard deviation (SD), median, minimum, and maximum. Summaries of change-from-baseline variables included only subjects with baseline values and corresponding value at the time point of interest. Categorical data included frequency and percentage. Medical Dictionary for Regulatory Activities version 21.1 was used for coding TEAEs. The overall incidence of TEAEs was displayed by system organ class (SOC), preferred term, and dose group. Vital signs measurements, ECG measurements, pure tone audiometric thresholds, and clinical laboratory test results were summarized using descriptive statistics by dose-group and time-point. The area under the plasma concentration-time curve, time 0–24 h post-dose (AUC_0–24_) and the area under the plasma concentration-time curve, time 0 to the last measurable non-zero concentration (AUC0-t), were calculated by the linear trapezoidal method. Maximum observed concentration (Cmax) and time to reach Cmax (tmax) were also calculated. If the maximum value occurs at more than 1 time point, tmax is defined as the first time point with this value. Apparent first-order terminal elimination half-life (T1/2) was calculated as 0.693 divided by the elimination-rate constant.

## Results

### Pharmacokinetics

**Fig. 2:**
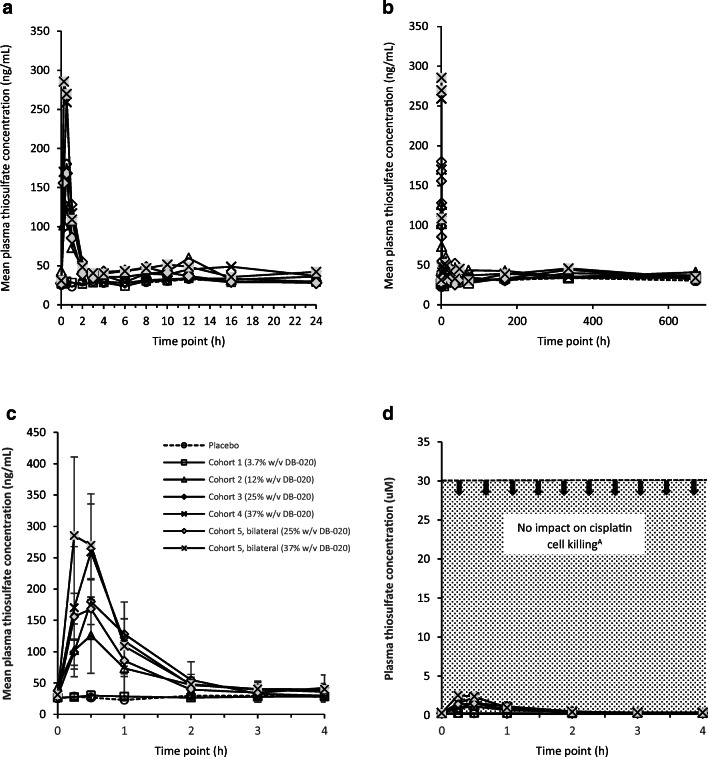
Human plasma thiosulfate levels following DB-020 IT administration. (**a**) Human plasma thiosulfate levels over 0 to 24 h following IT administration of DB-020, cohorts 1–5. (**b**) Human plasma thiosulfate levels over 0 to 672 h (28 days) following IT administration of DB-0202, cohorts 1–5. (**c**) Human plasma thiosulfate levels over 0 to 4 h following IT administration of DB-020, cohorts 1–5. (**d**) Human plasma thiosulfate levels over 0 to 4 h following IT administration of DB-020, cohorts 1–5 in relation to the concentration of thiosulfate that should have no impact on cisplatin cell killing (ie 30 uM)

90 subjects were screened and 42 healthy men (*n* = 17, 40%) and women (*n* = 25, 60%) aged 19 to 47 years were randomized and completed the trial. Following unilateral IT DB-020 administration in Cohorts 1 through 4 at concentrations of 3.7, 12, 25 and 37% *w*/*v* DB-020, no significant increase in thiosulfate levels above endogenous (placebo) levels were observed for Cohort 1, while minimal increases in maximum plasma thiosulfate levels were observed in Cohorts 2 through 4. The Tmax occurred approximately 30 min following DB-020 IT administration (Fig. 2), indicating rapid absorption/permeation following IT administration to the middle ear. No significant increase in endogenous plasma thiosulfate levels were observed for the remainder of the PK evaluation period (672 h) indicating the plasma thiosulfate levels are a result of rapid membrane permeability into the inner ear tissue, including cochlea, following IT DB-020 administration and not due to re-absorption/permeability of DB-020 elsewhere in eustachian tube or alimentary canal during elimination. The average half-life for plasma thiosulfate returning to endogenous levels was approximately 0.8 h (48 min) for cohorts 2–4 and thiosulfate levels returned to endogenous levels by approximately 2 h post administration. This indicates rapid clearance of thiosulfate back to endogenous levels after Cmax and is consistent with literature reports of thiosulfate pharmacokinetics in humans [19].

Following bilateral 25% and 37% *w*/*v* DB-020 IT administration (Cohort 5), plasma thiosulfate Cmax was similar to that following unilateral DB-020 administration at the same dose levels. There was an increase in exposure following bilateral DB-020 administration relative to unilateral. Plasma thiosulfate versus time profiles would be predicted to show either 2 distinct peaks, offset by approximately 30 min, with similar Cmax values or, if PK time points are limited, a plateau profile with similar maximum thiosulfate concentration at 2 adjacent time points offset by approximately 30 min. A plateau PK profile was observed following bilateral DB-020 IT administration consistent with predictions. The increase in exposure (AUC0–24) for 25% *w*/*v* DB-020 bilateral was approximately 1.5-fold which would be predicted based on twice the amount of DB-020 being administered. The increase in exposure for 37% w/v DB-020 bilateral was approximately 2.0-fold.

While dose cohorts 2–5 showed a slight increase in exposure in subjects receiving active drug relative to the endogenous levels of thiosulfate in placebo subjects, there was no significant dose relationship. A probable explanation for these results is the nominal and short-lived increase above endogenous levels for dose cohorts 2–5 do not contribute significantly to overall plasma thiosulfate exposure. The maximum thiosulfate concentration above endogenous level in Cohorts 2–5 ranged from 0.80 to 2.45 μM. These concentrations are predicted to be approximately an order of magnitude lower than plasma thiosulfate levels able to impact cisplatin anti-tumor efficacy based on preclinical in vitro tumor cell antiproliferation studies [12]. Therefore, as DB-020 IT administration is expected to precede cisplatin administration (1–3 h prior to cisplatin) and based on observed plasma thiosulfate PK following IT DB-020 administration, thiosulfate levels would be returning to or at endogenous levels by the time of cisplatin administration. Figure 2D shows plasma thiosulfate levels in relation to the thiosulfate concentration (30 uM) that should have no impact on cisplatin cancer cell killing abilities.

### Adverse events

Of the 42 subjects that were randomized and completed the study, 33 were exposed to DB-020: 6 subjects each were exposed to a total dose of 19 mg, 62 mg, and 124 mg DB-020, respectively; 7 subjects were exposed to a total dose of 186 mg, and 4 subjects each were exposed to a total dose of 248 mg and 372 mg. Nine additional subjects were treated with placebo.

There were no serious treatment-emergent adverse events (TEAE), study drug-related serious TEAEs, discontinuations due to TEAEs, or deaths in the study (Table 1). Five out of six subjects (83.3%) in the DB-020 62 mg unilateral treatment group and all subjects (100%) in the other treatment groups experienced at least 1 TEAE. All subjects in the placebo bilateral, 19 mg, 124 mg, and 186 mg DB-020 unilateral groups and the 124 and 186 mg DB-020 bilateral treatment groups experienced at least 1 study drug-related TEAE (Table 2). Seven of 8 subjects (87.5%) in the placebo unilateral and 5 of 6 subjects (83.3%) in the 62 mg DB-020 unilateral treatment group experienced at least 1 study drug related TEAE.

**Table 1:** Overall summary of Treatment-emergent Adverse Events (safety analysis set)

	All Placebo (Unilateral) (*n* = 8) S(%)E	All Placebo (Bilateral) (*n* = 1) S(%)E	DB-020 19 mg (Unilateral) (*n* = 6) S(%)E	DB-020 62 mg (Unilateral) (n = 6) S(%)E	DB-020 124 mg (Unilateral) (n = 6) S(%)E	DB-020 186 mg (Unilateral) (n = 6) S(%)E	DB-020 124 mg (Unilateral) (*n* = 4) S(%)E	DB-020 186 mg (Unilateral) (*n* = 5) S(%)E
At Least 1 TEAE	8(100%)36	1(100%)2	6(100%)26	5(83.3%)22	6(100%)29	6(100%)40	4(100%)37	5(100%)47
At Least 1 Severe TEAE	0	0	0	0	0	2(33.3%)7	0	0
At Least 1 Study	7(87.5%)	1(100%)2	6(100%)	5(83.3%)	6(100%)	6(100%)	4(100%)	5(100%)
Drug-Related TEAE^a^	25		15	13	23	35	31	43
At Least 1 Serious TEAE	0	0	0	0	0	0	0	0
At Least 1 Serious Study Drug-Related TEAE	0	0	0	0	0	0	0	0
Discontinuations	0	0	0	0	0	0	0	0
Deaths	0	0	0	0	0	0	0	0

Abbreviations: S = subject; E = event; TEAE = treatment-emergent adverse event. Study drug related was defined as, possibly, probably, or definitely related to study drug

**Table 2:** Study Drug-related Treatment-emergent Adverse Events of Any Grade Observed in at Least 20% of Any One Group (Safety Analysis Set)

	All Placebo (Unilateral) (n = 8) S(%)	All Placebo (Bilateral) (n = 1) S(%)	DB-020 19 mg (Unilateral) (*n* = 6) S(%)	DB-020 62 mg (Unilateral) (*n* = 6) S(%)	DB-020 124 mg (Unilateral) (n = 6) S(%)	DB-020 186 mg (Unilateral) (n = 6) S(%)	DB-020 124 mg (Unilateral) (*n* = 4) S(%)	DB-020 186 mg (Unilateral) (*n* = 5) S(%)
Subjects with at least 1 related TEAE	7(87.5%)	1(100%)	6(100%)	5(100%)	6(100%)	6(100%)	4(100%)	5(100%)
Ear and labyrinth disorders								
Ear congestion	5(62.5%)	0	6(100%)	4(66.7%)	5(83.3%)	2(33.3%)	2(50.0%)	3(60.0%)
Ear pain	3(37.5%)	1(100%)	2(33.4%)	1(16.7%)	6(100%)	5(83.3%)	4(100%)	5(100%)
Tinnitus	3(37.5%)	0	1(16.7%)	1(16.7%)	3(50.0%)	4(66.6%)	1(25.0%)	3(60.0%)
Vertigo	1(12.5%)	0	0	0	2(33.3%)	5(83.3%)	1(25.0%)	2(40.0%)
Ear discomfort	2(25.0%)	1(100%)	0	0	1(16.7%)	0	2(50.0%)	3(60.0%)
Ear pruritus	0	0	0	0	1(16.7%)	0	2(50.0%)	0
Noninfective myringitis	0	0	0	0	0	3(50.0%)	0	0
Hypoacusis	0	0	0	0	0	0	2(50.0%)	0
Nervous system disorders								
Headache	2(50.0%)	0	1(16.7%)	0	0	2(33.3%)	3(75.0%)	2(40.0%)
Dizziness	4(50.0%)	0	0	2(33.3%)	0	0	3(75.0%)	2(40.0%)
Hypoaesthesia	0	0	0	0	0	0	1(25.0%)	0
Dysgeusia	0	0	0	0	0	0	0	1(20.0%)
Gastrointestinal disorders								
Nausea	0	0	0	1(16.7%)	0	2(33.3%)	2(50.0%)	3(36.0%)
Vomiting	0	0	0	1(16.7%)	0	2(33.3%)	0	0
Dysphagia	0	0	0	0	0	0	0	1(20.0%)
Oral pain	0	0	0	0	0	0	0	1(20.0%)
General disorders and administration site conditions								
Facial pain	0	0	0	0	0	1(16.7%)	1(25.0%)	0
Respiratory, thoracic and mediastinal disorders								
Throat irritation	0	0	0	0	0	1(16.7%)	1(25.0%)	1(20.0%)
Oropharyngeal pain	0	0	0	0	0	0	0	1(20.0%)
Sinus pain	0	0	0	0	0	0	1(25.0%)	0
Musculoskeletal and connective tissue disorders								
Pain in jaw	0	0	0	0	0	0	0	1(20.0%)

Abbreviations: S = subject; MedDRA = Medical Dictionary for Regulatory Activities; TEAE = treatment-emergent adverse event Study drug-related was defined as possibly, probably, or definitely related to study drug. Note: Subjects were counted once if the same event occurred more than 1 time Note: This table presents the number of subjects with an event and the percentage of total subjects with an event. Percentages for each relationship category within a preferred term were based on the number of subjects with an adverse event in the preferred term. All other percentages were based on the total number of treated subjects Note: Adverse events coded according to MedDRA Version 21.1

**Table 3:** Dose levels per treatment cohort (active drug)

Cohort	# of Subjects	% Solution (weight/volume)	Molarity	mg/mL	mg
1 (unilateral)	6	3.7	0.15	37	19
2 (unilateral)	6	12	0.5	124	62
3 (unilateral)	6	25	1.0	248	124
4 (unilateral)	6	37	1.5	372	186
5 (bilateral)	4	25	1.0	248	124
	5	37	1.5	372	186

DB-020 concentrations of 12% and 25% and 37% w/v were used in this study. Percentage *w*/*v* and relevant unit conversions are presented in this table

Study drug-related TEAEs were clustered in the Ear and Labyrinth disorders, Nervous system disorders, and Gastrointestinal disorders system organ classes (SOCs). Study drug-related TEAEs occurring in at least 20% of subjects in any one group included ear congestion, ear pain, tinnitus, vertigo, ear discomfort, ear pruritus, noninfective myringitis, hypoacusis, headache, dizziness, hypoaesthesia, dysgeusia, nausea, vomiting, dysphagia, oral pain, facial pain, throat irritation, oropharyngeal pain, sinus pain, and pain in jaw (Table 2). These events were consistent with TEAEs observed with general administration of solutions to the inner ear via IT injections [20]. There were no clear trends for increasing rates of TEAEs with increasing doses of DB-020.

Two of 6 subjects (33.3%) in the 186 mg DB-020 unilateral cohort experienced severe TEAEs including vertigo, nausea and vomiting. All other TEAEs were assessed as mild or moderate. Both subjects received IV antiemetics and hydration and the severe TEAEs resolved within 24 h. To mitigate the caloric response, a possible cause of these TEAEs, the syringes were warmed in a water bath prior to administration to Cohort 5 subjects. No further severe vertiginous events were seen and all other TEAEs were mild or moderate. There were no clinically significant changes from baseline in laboratory parameters, electrocardiograms, vital signs, or physical examinations. One subject in the bilateral dose group (Cohort 5) refused the administration of the second administration of DB-020 due to feeling pain with the first injection. This subject completed all visits in the study.

### Audiometric tests

Changes from Screening in air conduction (AC) pure tone thresholds were analyzed on Day 3, Day 8, Day 15, and Day 29 post-IT injection.

Median threshold changes within each cohort at Day 29 were within 5–10 dB of screening at all test frequencies for ears injected with DB-020 or placebo. Variability in the extended high frequencies was similar in all cohorts however, some subjects had greater threshold variability at certain frequencies at intermediate visits. Measurements were taken at two different test sites: one used at screening and Day 29, the other at intermediate visits which likely contributed to the variability seen at frequencies >8 kHz. There was no clear pattern of change from baseline in bone conduction frequency thresholds from 250 to 4000 Hz between ears with increasing dose of DB-020 or time after drug administration.

There was no change from baseline in intrusiveness, sense of control, cognitive, sleep, auditory, relaxation, quality of life, emotional, or tinnitus functional index total scores in Cohorts 1, 2, and placebo after drug administration. One subject in Cohort 3, one in Cohort 4, and one in Cohort 5 had temporary increases of mean TFI score on Day 8 to a “small problem” (18–31 range/100) by TFI definition which resolved by Day 15.

There was no clear pattern of change in Hearing Handicap Inventory for Adults (HHIA) social, emotional, or total scores in Cohorts 1, 2, and placebo after drug administration. One subject in Cohort 3, 4, and 5 respectively experienced temporary “mild-moderate handicap” on Day 8, which resolved by Day 15.

## Discussion

Cisplatin is widely used for the treatment of certain solid tumors. Ototoxicity is a known serious side effect of cisplatin often leading to permanent hearing loss. In order to mitigate this side effect, adjustments away from optimal cisplatin dose levels or discontinuation of cisplatin treatment, with potential negative impact on cancer outcomes, may be applied by oncologists. To date no treatment exists to prevent or treat cisplatin-induced hearing loss.

DB-020 Injection is a novel formulation comprised of sodium thiosulfate pentahydrate in sodium hyaluronate to be delivered via IT injection within three hours prior to cisplatin administration. Thiosulfate has been shown to permanently bind to cisplatin and render cisplatin nontoxic thereby protecting OHC and hearing loss. Nonetheless, systemic administration of thiosulfate has only produced modest effects on hearing protection. This is likely due to the high concentration of systemic thiosulfate necessary to achieve high cochlear levels which prevent a timely administration proximal to cisplatin infusion without negatively impacting the cancer treatment.

To date, a main uncertainty has been whether thiosulfate administered IT can provide adequate cochlear exposure in relation to the time of systemic cisplatin treatment in humans to maximize hair cell protection in the inner ear without negatively impacting its efficacy toward cancer progression. IT injection of DB-020 allows delivery of high concentration of thiosulfate to the cochlea prior to cisplatin administration in humans with only nominal and temporary systemic increase of thiosulfate levels. Protection in guinea pigs was seen at all frequencies which strongly suggests good DB-020 permeability, not necessarily limited to the round window. Similarly, it was observed that volume increase, while keeping DB-020 concentration constant, resulted in increased thiosulfate cochlear exposure suggesting additional access routes beyond the round window membrane. The selected concentration of DB-020 and route of administration will likely facilitate permeability through the round window, oval window and potentially other routes as indicated by human plasma PK results suggesting rapid permeation of thiosulfate through the middle ear (Fig. 2).

Maximum thiosulfate plasma concentration observed in this Ph1 study was approximately 10-fold lower than the thiosulfate levels expected to impact cisplatin activity after clinically relevant doses, even at the highest DB-020 concentration and after bilateral administration. Further, IT administration of DB-020 is expected to be 1 to 3 h prior to cisplatin dosing. Thus, based on the observed plasma thiosulfate PK following DB-020 administered IT to humans up to the maximum feasible dose (37% *w*/*v*), thiosulfate levels would be returning to or at endogenous levels by the time of cisplatin administration. These results provide the rationale for why DB-020 is not expected to interfere with cisplatin anti-tumor efficacy.

Furthermore, DB-020 was shown to be safe and well tolerated in healthy volunteers at all dose levels tested. Most TEAEs reported in the study were generally mild to moderate, transient and likely related to the IT administration procedure. No serious adverse events (AE) were reported, no discontinuations to the treatment and no deaths occurred during the study.

Two subjects receiving 186 mg of DB-020 experienced severe vertigo, nausea and vomiting. These events had short duration (less than a day) and subsided upon administration of IV antiemetics and hydration. A safety review committee carefully examined all safety data available for this cohort post treatment and reached the conclusion that the likely cause of these severe events was a caloric response which has been well described in subjects receiving IT injections [21]. To mitigate TEAEs due to caloric response, the syringes were warmed in a water bath prior to administration to newly enrolled subjects and no further severe vertiginous events occurred.

Overall, DB-020 appeared safe and well tolerated at each dose level tested. TEAEs were consistent with events associated with IT delivery and no clinically significant changes from baseline in laboratory assessments, electrocardiograms (ECG), vital signs, or physical examinations were observed. There were no significant permanent changes in tympanometry, distortion product otoacoustic emissions, median thresholds on air and bone conduction testing, and otoscopy results. These promising results led to the expansion of the clinical program with the initiation of a randomized clinical trial with DB-020 (DB-020-002) in cancer patients undergoing cisplatin treatment to evaluate the safety, tolerability and efficacy of DB-020 in preventing ototoxicity and subsequent hearing loss.
